# Impacts of Modification of Alloying Method on Inclusion Evolution in RH Refining of Silicon Steel

**DOI:** 10.3390/ma10101206

**Published:** 2017-10-19

**Authors:** Fangjie Li, Huigai Li, Shaobo Zheng, Jinglin You, Ke Han, Qijie Zhai

**Affiliations:** 1 State Key Laboratory of Advanced Special Steel, Shanghai Key Laboratory of Advanced Ferrometallurgy, School of Materials Science and Engineering, Shanghai University, Shanghai 200072, China; fangjieli119@126.com (F.L.); sbzheng@staff.shu.edu.cn (S.Z.); jlyou@163.com (J.Y.); qjzhai@shu.edu.cn (Q.Z.); 2 National High Magnetic Field Laboratory, Florida State University, Tallahassee, FL 32310, USA; han@magnet.fsu.edu

**Keywords:** silicon steel, RH refining, inclusions, evolution

## Abstract

This study explores the effect of introducing additional alloy elements not only in a different order but also at different stages of the Ruhrstahl-Heraeus (RH) process of low-carbon silicon steel production. A more economical method, described as “pre-alloying”, has been introduced. The evolution of MnO-FeO inclusions produced by pre-alloying was investigated. Results show that spherical 3FeO·MnO inclusions form first, then shelled FeO·zMnO (z = 0.7–4) inclusions nucleate on the surface of pre-existing 3FeO·MnO. Spherical FeO·zMnO (z = 3–5) is further evolved from shelled 3FeO·MnO by diffusion. Because these MnO-FeO inclusions float up into the slag before degassing, the pre-alloying process does not affect the quality of the melt in the end. Both carbon content and inclusion size conform to industry standards.

## 1. Introduction

Silicon steel is a soft magnetic material that is widely used in the manufacture of electrical machinery, motors, and transformers [1]. Production of this low-carbon steel requires careful control of the oxide inclusions that occur during the steelmaking process [2,3,4]. One commonly used technique for refining low-carbon steels is the Ruhrstahl-Heraeus (RH) process [5,6,7]. The RH refining process has three stages: (1) decarburization, (2) deoxidization and alloying, and (3) degassing. During the first stage, oxygen is blasted into the melt to keep the carbon level low. The second stage has two aims: (1) to bring the oxygen level down before the processing continues, and (2) to ensure the proper combination of the additional elements introduced to form a new alloy. The third stage also has two aims: (1) to remove the hydrogen and nitrogen that had been naturally absorbed from the air during treatment prior to the RH process, and (2) to allow inclusions to float up into the slag.

During deoxidization and alloying, the second stage, aluminum (Al) and ferrosilicon (FeSi) are frequently used as deoxidizers. When these alloys are added separately, they produce either Al_2_O_3_ or SiO_2_ inclusions. Zhen et al. pointed out that slabs produced by Al deoxidization are inferior in quality to those produced by Si deoxidization [8]. This may be in part because SiO_2_ inclusions (produced by Si deoxidization) are less likely to cluster than Al_2_O_3_ inclusions (produced by Al deoxidization) [9]. When FeSi is added first, the resulting SiO_2_ inclusions combine with any Al that is subsequently added, but the reverse does not occur. The behavior of inclusions in melts varies, depending on the sequence of alloy addition [10].

After deoxidization, conventional alloying occurs, not only with Si and Al but also with Mn. As an alloying agent, low-carbon ferromanganese (FeMn) is as effective as pure Mn but much more economical, so it is often used in this stage. In the current study, medium-carbon FeMn, which is even less expensive than low-carbon FeMn, was introduced into the melt in the first stage, that is, before the beginning of decarburization. This fabrication method is referred to in this study as “pre-alloying”. The disadvantage of pre-alloying is that Mn oxide inclusions begin immediately to form and build up in the melt. The countervailing advantage, however, is that the Mn that remains after oxides are formed is available during the second stage to reduce the consumption of costlier low-carbon FeMn. Previous studies have analyzed the manganese balance between slag and steel in the pre-alloying process from both thermodynamics and dynamics [11,12,13]. The yielding rate of FeMn and its influencing factors has also been investigated [14]. Nevertheless, the evolution of inclusions in silicon steel after introducing the pre-alloying procedure has rarely been studied. One of the objectives of this study was to investigate the growth and behavior of the Mn oxide inclusions produced by the early introduction of medium-carbon FeMn.

The third stage of the RH process is a procedure named “degassing”. Degassing occurs when argon is blasted into the melt, causing inclusions to float up into the slag. This process is important for removing the silicon oxide and aluminum oxide inclusions that form during the deoxidization and alloying stage [15]. Zhang et al. claimed that the smaller inclusions collide with one another to form larger inclusions, which then float up to the slag where they are absorbed and thus removed from the melt [16].

The aim of this work is to investigate the impacts of modification of the alloying method on the inclusion evolution in RH refining of silicon steel, with an emphasis on the composition and size of the inclusions. The carbon content induced by medium-carbon FeMn addition in pre-alloying was also probed to make sure it met the industry expectation.

## 2. Experimental Procedure

**Figure 1:**
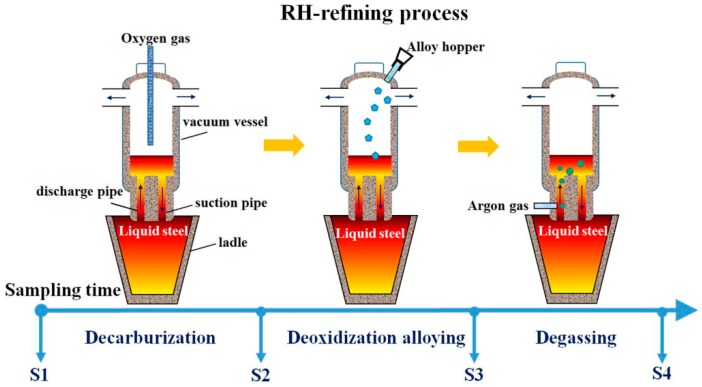
Illustration of sampling during the RH (Ruhrstahl-Heraeus) refining process.

RH refining was processed in a 160-t ladle with MgO-C-type lining refractory. The molten steel in the ladle undergoes the processes of decarburization, deoxidization alloying, and degassing to obtain a semi-finished product. Samples were taken by drawing 3 kg of steel melt from the industrial ladle at four key states of RH refining. The first drawing occurred before the start of decarburization but after medium-carbon FeMn alloy had been added; the second after decarburization had been completed; the third after FeSi and Al had been introduced for deoxidization/alloying, followed by the addition of low-carbon FeMn for further alloying; and the fourth after degassing had been completed (See Figure 1). The four samples were investigated to clarify the formation and evolution of inclusions, not only in the pre-alloying process but also in the deoxidization and alloying process.

**Figure 2:**
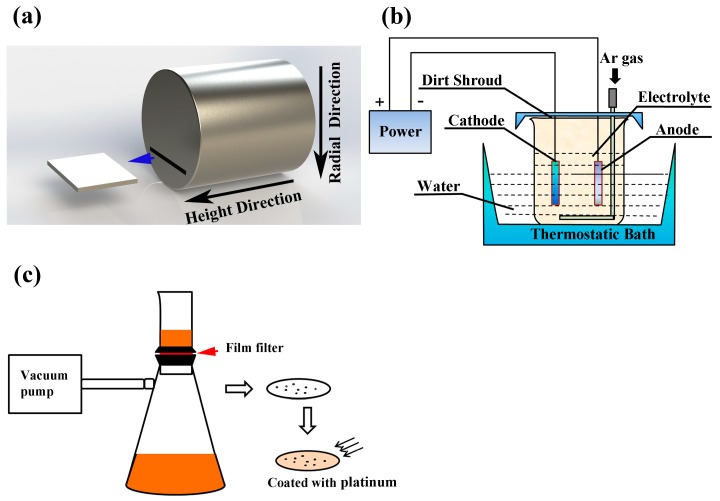
(**a**) Sheet cut from the ingot at the halfway point of the radius (“Height direction” and “Radial direction” are the vertical and horizontal directions of the RH ladle, respectively). (**b**) Schematic of non-aqueous solution electrolysis of a steel sample. The anode is a sheet cutting from the sample and the cathode is a platinum plate. (**c**) Collection of inclusions separated by vacuum filtration.

Each drawing was cooled down to room temperature and allowed to solidify into a cylindrical ingot. From each ingot, a sample was cut in the form of a thin sheet (50 × 40 × 2 mm^3^), taken from the longitudinal plane at the halfway point of the radius (see Figure 2a). Each of these four sample sheets (S1, S2, S3, S4) was then electrolyzed in a non-aqueous solution composed of methanol, maleic anhydride, and tetramethyl ammonium chloride [17]. After 6 hours of electrolysis, a good portion of the steel matrix had dissolved into the non-aqueous solution. When this solution was passed through a polycarbonate membrane film filter (open pore size 3 μm), the oxide inclusions from the sample sheet were deposited on the filter (see Figure 2c) [18]. This filter was coated in platinum to improve conductivity, and inclusions were then examined in a scanning electron microscope (SEM, Hitachi SU 1510, Hitachi, Tokyo, Japan) with an energy-dispersive X-ray spectrometer (EDS, Hitachi, Tokyo, Japan). Fifty inclusions from each filter were randomly selected for statistical analysis.

## 3. Results and Discussion

### 3.1. Analysis of Oxide Inclusions in RH Refining

**Figure 3:**
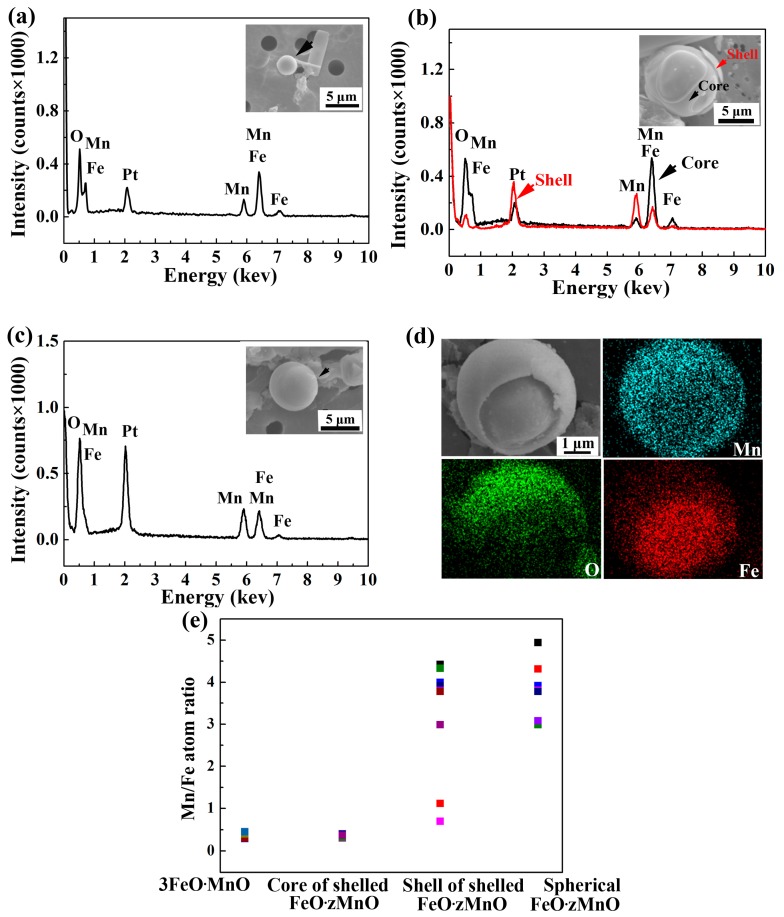
(**a**) Morphology (black arrow) and EDS spectrum of the spherical inclusions typical of S1. The Pt peak is derived from the platinum plating that is applied to the inclusions before analysis. On average, S1 inclusions are smaller than S2 inclusions. (**b**) Morphology and EDS spectrum of the inner core (black arrows) and outer shell (red arrows) of the shelled inclusions that occur in S2. (**c**) Morphology and EDS spectrum of the unshelled spherical inclusions that also occur in S2. (**d**) Mapping analysis of the distribution of Mn, O, and Fe elements in shelled inclusions. Note that the intensity of Mn and O elements is high in the outer area (shell) and weak in the inner area (core), but that of Fe is high in the core and weak in the shell. (**e**) Composition analysis of MnO-FeO oxide inclusions in S1 and S2. The first two columns on the left show that the Mn/Fe ratio for all S1 inclusions (calculated to be 0.36 ± 0.09) is at the same level as for the core of shelled inclusions in S2 (0.35 ± 0.05). The last two columns on the right show that the Mn/Fe ratio for S2 spherical inclusions (average of 4 with maximum 5 and minimum 3) is at the same level as for the shells alone in the S2 shelled inclusions (average of 2 with maximum 4 and minimum 0.7), and the shell has a wider ratio range from 0.7 to 4.

Inclusions in sample S1 were analyzed using EDS and found to be composed of a MnO-FeO oxide rich in Fe, which was called 3FeO·MnO according to the average atomic ratio calculated from the EDS results (see Figure 3a,e). These inclusions were photographed using SEM and were found to be spherical in all cases.

The images of inclusions in sample S2 were more complex. These inclusions appeared in two separate spherical forms, one with a shell and the other without (see Figure 3b,c). EDS showed that these two forms were distinct from one another. The unshelled form was composed entirely of Mn-rich oxide, which was identified as FeO·zMnO. The average value of z calculated from EDS data is close to 4 (Figure 3e). In the shelled form, the outer shell was also composed of FeO·zMnO, but the average value of z is close to 2. The inner core, however, was composed of 3FeO·MnO, the same composition as in the whole sphere of the S1 inclusions (see Figure 3d,e).

Above results indicate that the spherical 3FeO·MnO forms a molten-glass-like product before the melt reaches the solidification temperature and later acts as a heterogeneous nucleation site for shelled inclusions. Dissolved Mn in the melt diffuses towards 3FeO·MnO to nucleate FeO·zMnO on 3FeO·MnO. At this stage, such oxides form molten-glass-like products made of FeO·zMnO shell and 3FeO·MnO core. The solidification temperature of FeO-MnO increases with the increase of MnO content [19]. Because FeO·zMnO has a higher MnO content and hence a higher solidification temperature than 3FeO·MnO, FeO·zMnO solidifies first, forming the shell. Subsequently, 3FeO·MnO, with its lower solidification temperature, forms the solid core, leaving a distinct shell-core structure in a solid state.

**Figure 4:**
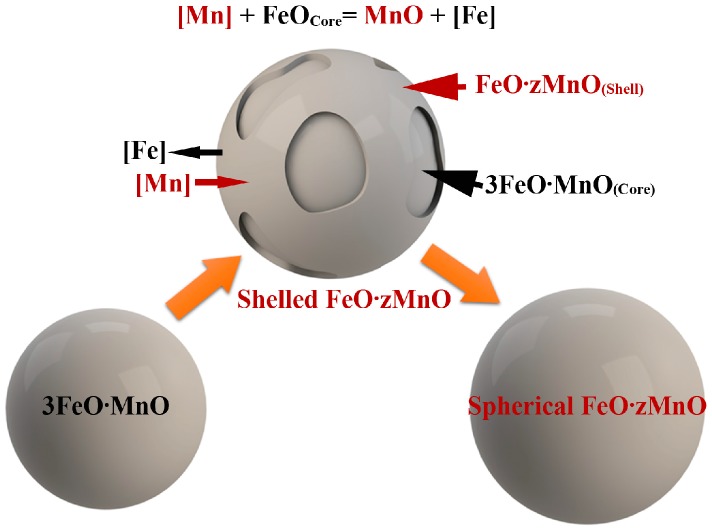
Schematic illustration of the evolution of MnO-FeO inclusions. Spherical 3FeO·MnO (left-hand) forms a molten-glass-like product before the melt reaches the solidification temperature. The center sphere is a shell-core inclusion formed by dissolved Mn diffusing towards 3FeO·MnO to nucleate FeO·zMnO shell on 3FeO·MnO. 3FeO·MnO forms the solid core after solidification, leaving a distinct shell-core structure.

In sample S2, the ratio (z) of Mn/Fe in FeO·zMnO varies from 0.7 to 4 in shelled inclusions and from 3 to 5 in spherical FeO·zMnO. When z = 0.7, the composition is close to 2FeO·MnO. When z = 4, the composition is FeO·4MnO. This indicates that inter-diffusion occurred within the inclusions. The higher chemical potential of Mn in the oxide shell of S2 inclusions may have resulted in the diffusion of Mn from the shell into the inner core. Conversely, the higher Fe content in the core diffuses into the shell. When diffusion leads to z > 3, a distinct interface between shell and core disappears and spherical FeO·zMnO forms (see Figure 4).

**Figure 5:**
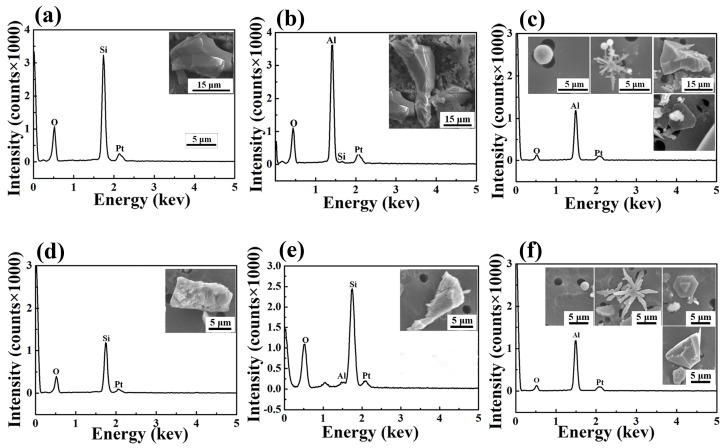
The type and three-dimensional images of Mn-Free oxides in S3 and S4 (**a**) SiO_2_ in S3, (**b**) Al-Si oxide in S3, and (**c**) Al_2_O_3_ in S3; (**d**) SiO_2_ in S4, (**e**) Al-Si oxide in S4, and (**f**) Al_2_O_3_ in S4.

When samples S3 and S4 were examined, three different types of Mn-free oxide inclusions had emerged: SiO_2_, Al-Si oxide, and Al_2_O_3_ (see Figure 5). No MnO-FeO oxides were found either at the beginning or the end of the third and final (degassing) stage, despite the fact that FeMn had been added not only in the second stage (traditionally reserved for deoxidization and alloying) but also in the first stage (traditionally reserved only for decarburization). This means that the pre-alloying process had not interfered with the level of inclusions in the final product.

**Figure 6:**
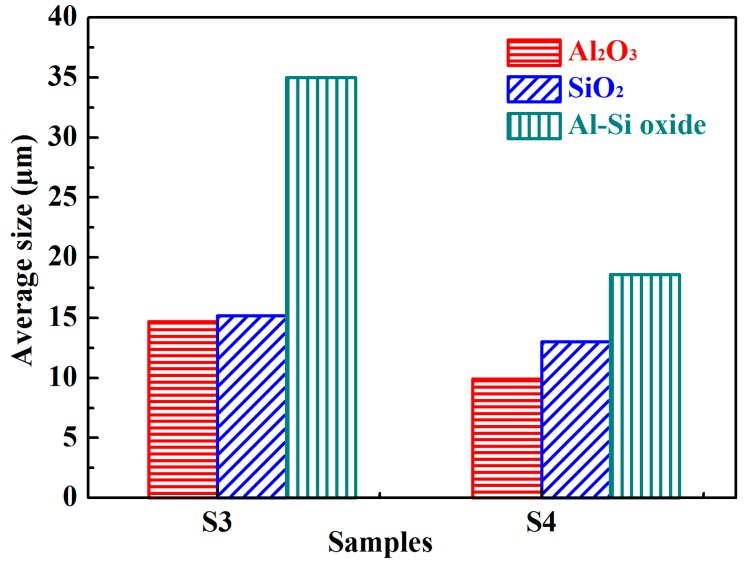
The average sizes of oxide inclusions in the S3 and S4 samples.

A decrease in size occurred between the second and third stages in all types of oxide inclusions (see Figure 6), because the larger inclusions had already floated into the slag.

### 3.2. Quality and Cost Analysis of the Pre-Alloying Innovation

Carbon content is an important factor in the production of low-carbon silicon steel. The carbon content in the first stage of the conventional alloying process is in the range of 3.0–4.0 × 10^−2^ wt. % for high-quality silicon steel [20,21]. Because of the early addition of medium-carbon FeMn alloy in the pre-alloying process, the carbon content at the end of first stage was considerably higher than the level required for high quality steel. The carbon content dropped sharply during the second stage and reached 1.0 × 10^−3^ wt. % at the end of RH refinement, a level even lower than that produced in the conventional alloying process (see Table 1). Thus, the carbon content can be expected to consistently meet or exceed the industry standard for high quality silicon steel.

Because MnO-FeO oxides form immediately after the addition of low-cost, medium-carbon FeMn in the pre-alloying process, part of the added Mn element (about 21%) goes into inclusions during the first stage. This loss of Mn is compensated by the later addition of high-cost, low-carbon FeMn during the second stage. The amount of high-cost FeMn alloy needed in the second stage is thus reduced about 20%, by the previous addition of low-cost, medium-carbon FeMn in the first stage. Based on the price of the two kinds of FeMn alloys in 2017 [22,23], the overall cost reduction introduced by pre-alloying amounts to 3%.

## 4. Conclusions

A new fabrication method of pre-alloying was introduced in the RH refining process of low-carbon silicon steel. The evolution of inclusions produced by pre-alloying was investigated. The quality of the final product of RH refining and the cost of the pre-alloying RH process were analyzed. Three main conclusions were obtained:

(1) MnO-FeO inclusions formed as a by-product of pre-alloying. The evolution of the oxide is as follows: spherical Fe-rich 3FeO·MnO becomes shelled Fe-rich, low-melting point 3FeO·MnO in the core and Mn-rich, high-melting point FeO·zMnO on the surface of the shell. Some of shelled inclusions become spherical Mn-rich FeO·zMnO. These inclusions are eliminated at the beginning of the degassing stage.
(2) Inclusion evolution in pre-alloying RH refining is different from that in conventional RH refining, but the ingots produced by pre-alloying are of a quality that meets or exceeds industry-wide specifications with respect to quality, carbon content, and inclusion size.
(3) When pre-alloying is used, the amount of high-cost, low-carbon FeMn alloy required during the deoxidization/alloying stage is reduced by 20%, thus reducing the overall cost of the process by 3%.

## Figures and Tables

**Table 1:** Chemical compositions of steel samples (wt. %) in different stages of the pre-alloying process and in the final stage of the conventional alloying process.

Process	Point	C	Si	Mn	Al_s_
Pre-alloying process	S1	4.4 × 10^−2^	2.1 × 10^−3^	6.1 × 10^−2^	2.0 × 10^−3^
	S2	1.0 × 10^−3^	2.3 × 10^−3^	4.9 × 10^−2^	1.9 × 10^−3^
	S3	5.0 × 10^−3^	2.6 × 10^−1^	3.0 × 10^−1^	3.3 × 10^−1^
	S4	1.0 × 10^−3^	2.6 × 10^−1^	3.6 × 10^−1^	2.8 × 10^−1^
Conventional alloying process	End of RH	<5.0 × 10^−3^	2.5 × 10^−1^	3.5 × 10^−1^	2.8 × 10^−1^
